# miR-125b regulates differentiation and metabolic reprogramming of T cell acute lymphoblastic leukemia by directly targeting A20

**DOI:** 10.18632/oncotarget.12018

**Published:** 2016-09-14

**Authors:** Zixing Liu, Kelly R. Smith, Hung T. Khong, Jingshan Huang, Eun-Young Erin Ahn, Ming Zhou, Ming Tan

**Affiliations:** ^1^ Mitchell Cancer Institute, University of South Alabama, Mobile, AL, USA; ^2^ Huntsman Cancer Institute, University of Utah, Salt Lake City, Utah, USA; ^3^ School of Computing, University of South Alabama, Mobile, AL, USA; ^4^ Cancer Research Institute, Central South University, Changsha, Hunan, China; ^5^ Department of Biochemistry & Molecular Biology, University of South Alabama, Mobile, AL, USA

**Keywords:** T cell, lymphocytic leukemia, differentiation, miR-125b, A20

## Abstract

T-cell acute lymphoblastic leukemia (T-ALL) is an aggressive hematopoietic malignancy. Although it has been reported that overexpression of miR-125b leads to T-ALL development, the underlying mechanisms of miR-125b action are still unclear. The goal of this study is to delineate the role of miR-125b in T-ALL development. We found that miR-125b is highly expressed in undifferentiated leukemic T cells (CD4-negative) while its expression is low in differentiated T cells (CD4-positive). Overexpression of miR-125b increased the CD4-negative population in T cells, whereas depletion of miR-125b by miR-125b-sponge decreased the CD4-negative cell population. We identified that A20 (TNFAIP3) is a direct target of miR-125b in T cells. Overexpression of miR-125b also increased glucose uptake and oxygen consumption in T cells through targeting A20. Furthermore, restoration of A20 in miR-125b-overexpressing cells decreased the CD4-negative population in T cell leukemia, and decreased glucose uptake and oxygen consumption to the basal level of T cells transfected with vector. In conclusion, our data demonstrate that miR-125b regulates differentiation and reprogramming of T cell glucose metabolism via targeting A20. Since both de-differentiation and dysregulated glucose metabolism contribute to the development of T-cell leukemia, these findings provide novel insights into the understanding and treatment of T-ALL.

## INTRODUCTION

In 2015, National Cancer Institute estimated there will be 54,270 new cases of leukemia (3.3% of all new cancer cases) and an estimated 24,450 people (4.1% of all cancer deaths) will die of this disease in the United States. There are about 4,000 new cases of acute lymphoblastic leukemia in the United States each year [1]. T-ALL (T-cell acute lymphoblastic leukemia) is an aggressive hematopoietic cancer with a five year survival rate about 50% [2]. A significant number of T-ALL patients show poor responses to treatment. Compared with the more common B-cell-lineage ALL, T-ALL is defined by distinct clinical and biological characteristics and is generally associated with more unfavorable clinical features [3]. T-ALL can be subdivided into different stages depending on different markers (CD3, CD7, CD28, CD1a, CD34, CD4 and CD8) expressed in the T cells. NOTCH1 and several microRNAs have been reported to play a key role in T cell differentiation and T-ALL development [2, 4–5]. However, the mechanisms of T-ALL development are still not fully understood.

A20, also known as TNF-α–induced protein 3 (TNFAIP3), is a zinc finger protein with both a ubiquitinase ligase domain and a deubiquitinase domain. A20 is a key protein in negative feedback inhibition of the nuclear factor kB (NF-κB) activation [6–7]. Deficiency of A20 results in constant activation of NF-κB in lymphocytes which leads to autoimmune diseases [8]. A20 is frequently found to be inactivated, mutated or deleted in leukemia [9–10]. B cells lacking A20 display impaired differentiation and hyperactivation [11].

MicroRNAs (miRNAs) are short ribonucleic acid (RNA) molecules which play key roles in many physiological and developmental processes by controlling gene expression. Deregulated expression of certain miRNAs is responsible for carcinogenesis [12]. We have reported that miR-125b plays an important role in cancer cell drug resistance and cancer stem cells enrichment [13–14]. miR-125b has also been reported to inhibit cell differentiation [15–18]. miR-125b is highly expressed in hematopoietic stem cells, and promotes lymphoid-fate decisions [19]. Overexpression of miR-125b in hematopoietic stem/progenitor cells leads to leukemia of both myeloid and lymphoid lineages in mice, indicating a critical function of miR-125b in early hematopoiesis [20]. In addition, miR-125 accelerates the leukemogeneisis induced by BCR-ABL [20]. However, the detailed molecular mechanism of miR-125b action in T-ALL development is poorly understood.

In this study, we demonstrate that miR-125b is highly upregulated in the undifferentiated cell population compared to the differentiated cell population, demonstrating its heterogeneous expression within T-ALL cells. Furthermore, our data showed that miR-125b plays a critical role in NF-kB signaling, glucose uptake and oxygen consumption via targeting A20, revealing the mechanism of miR-125b action in de-differentiation and metabolic reprogramming in T cells.

## RESULTS

### miR-125b regulates T cell differentiation

**Figure 1:**
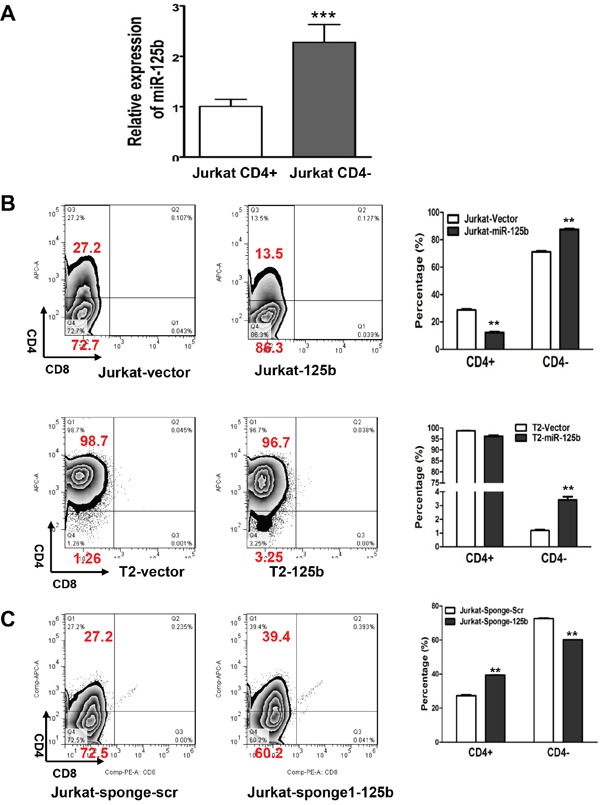
miR-125b regulates T cell differentiation **A.** qRT-PCR was performed to examine the expression of miR-125 in CD4+population and CD4-pouplation of Jurkat cell lines. RNU6B was used as an internal control and for normalization of the data. Columns represent the mean of three independent experiments; bars represent SE. ***, p<0.001. **B** and **C.** 1×10^6^ Jurkat-vector, Jurkat-miR-125b, T2-vector and T2-miR-125b stable cell lines were incubated with CD4 and CD8 antibodies for 45min, washed, and then the cells were analyzed by flow cytometry.

It has been reported that overexpression of miR-125b in early stage hematopoietic cells leads to leukemia in mice. In a recent study, approximately 50 percent of mice developed T-cell acute lymphoblastic leukemia (T-ALL) in miR-125b overexpressing mice [20]. However the mechanism on how miR-125b causes T-ALL is still elusive. To examine the function of miR-125b in T cell differentiation, CD4-positive (CD4+, mature T cells) and CD4-negative (CD4-, immature T cells) populations were sorted from leukemic T-cell line Jurkat cells by flow cytometry. Compared with CD4+ Jurkat cells, CD4- Jurkat cells showed significantly higher expression of miR-125b (Figure 1A). This inverse correlation of miR-125b with the expression of T cell marker CD4 suggests that miR-125b may inhibit T cell differentiation. To determine whether miR-125b regulates the differentiation of T cells, Jurkat cells and a more differentiated cell line T2 cells were transfected with a control pMSCV-vetor and a miR-125b expressing vector pMSCV-miR-125b (Figure 1B, Supplementary Figure 1) and then the expression of CD4 were measured in these cells. The result showed that the CD4- population in both Jurkat-miR-125b cells and T2-miR-125b cells was dramatically increased compared with the cells transfected with control vector alone. To further confirm these results, we blocked the function of miR-125b with a validated miRNA competitive inhibitor miR-125b sponge in Jurkat cells (Figure 1C) [20]. Depletion of miR-125b in Jurkat cells decreased the CD4 negative population. These results demonstrate that miR-125b inhibits the differentiation of T cells, which may contribute to the development of T-ALL.

### A20 is a direct target of miR-125b in T cell lymphocytic leukemia cells

**Figure 2:**
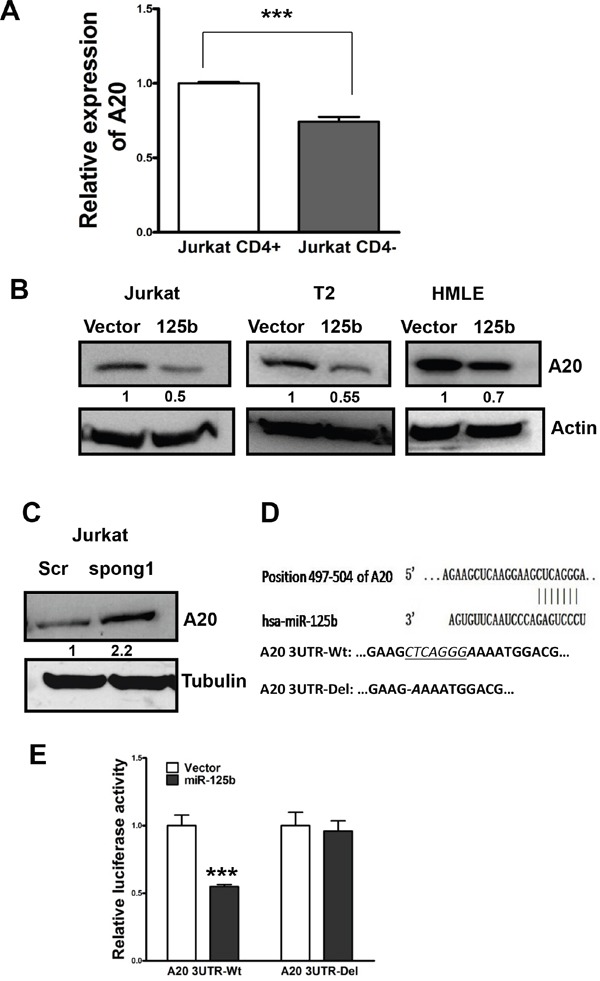
A20 is a direct target of miR-125b in T cells **A.** qRT-PCR was performed to examine the expression of A20 in CD4+population and CD4-pouplation of Jurkat cell lines. GAPDH was used as an internal control and for normalization of the data. Columns represent the mean of three independent experiments; bars represent SE. ***, p<0.001. **B** and **C.** Jurkat-vector, Jurkat-miR-125b, T2-vector, T2-miR-125b, HMLE-vector, HMLE-miR-125, Jurkat-scramble and Jurkat-miR-125b-spong1 stable cell lines were collected. Cell lysates were prepared for Western blotting with an antibody against A20 with β-actin used as a loading control. **D.** A20 3′ UTR contains a predicted miR-125b-binding site. Alignment between the miR-125b seed sequence and A20 3′ UTR is shown. A schematic diagram shows the wild type and mutant 3′UTR of A20. **E.** Jurkat cells were co-transfected with luciferase reporter plasmids with or without 3′-UTR of A20, pre-miR-125, or pre-miR-negative (Ctr). 48 hours post-transfection, cells were harvested and lysed with lysis buffer. Luciferase activity was measured by using a dual luciferase reporter assay. The pRL-TK vector was used as an internal control. The results were presented as relative luciferase activity (firefly LUC/Renilla LUC).

Because miR-125b is capable of inhibiting T cell differentiation from CD4- to CD4+, we searched miRNA databases for potential miR-125b targets that may contribute to T cell differentiation. The three public miRNA databases (TargetScan, Pictar, and MicroRNA) all predicted that A20 might be a target for miR-125b, because the 3′-UTR of A20 contains a highly conserved binding site from position 497 to 504 for miR-125b. To test whether miR-125 inhibits the expression of A20, we first compared the expression level of A20 by qRT-PCR in CD4- and CD4+ Jurkat cells. Compared with the high expression of A20 in CD4+ cells which express a low level of miR-125b, the expression of A20 is significantly lower in CD4- population which has high expression of miR-125b (Figure 2A, Figure 1A). To further confirm whether miR-125b could target A20, we forced ectopic expression of miR-125b into Jurkat, T2 and HMLE cells. Compared with cells transfected with vector alone, miR-125b overexpressing cells showed a dramatically lower expression of A20 (Figure 2B). Meanwhile, Jurkat cells with depletion of miR-125b by Spong1 have an increased expression of A20 in comparison with cells transfected with scramble control (Figure 2C). We further investigated whether miR-125b directly targets the 3′-UTR of A20 mRNA, and performed luciferase reporter analysis by co-transfecting a vector containing pMIR reporter-luciferase fused with the wild type (Wt) or miR-125b binding site deleted 3′-UTR of A20 mRNA and miR-125b or vector control. Overexpression of miR-125b decreased the luciferase activity of the reporter with 3′-UTR of A20 by about 40% in Jurkat cells (Figure 2D and 2E). However, no inhibitory effect of miR-125b on the activity of the reporter with mutant 3′-UTR of A20 was detected. Taken together, these results indicate that A20 mRNA is a direct target of miR-125b.

### miR-125b regulates CD4 differentiation in T cell lymphocytic leukemia cells through targeting A20

**Figure 3:**
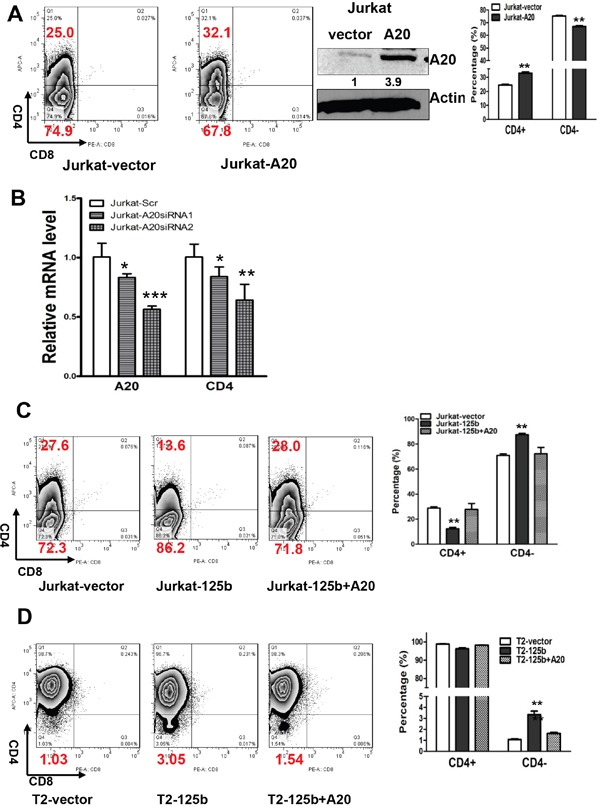
MiR-125 regulates CD4 differentiation marker in T-ALL cells through targeting A20 **A.** 1×10^6^ Jurkat-vector and Jurkat-A20 stable cell lines were incubated with CD4 and CD8 for 45min, washed, and then the cells were analyzed by flow cytometry. Jurkat-vector and Jurkat-A20 stable cell lines were collected. Cell lysates were prepared for Western blotting with an antibody against A20, and β-actin was used as a loading control. **B.** Jurkat cells were transfected with scrambled siRNA or A20 siRNAs. Forty-eight hrs after transfection, Cells were harvested and used for extracting RNA. qRT-PCR was performed to validate the expression of CD4 in Jurkat with depletion of A20 by siRNA. GAPDH was used as an internal control and for normalization of the data. Columns represent the mean of three independent experiments; bars represent SE. ***, p<0.001. **C** and **D.** 1×10^6^ Jurkat-vector and Jurkat-miR-125b, Jurkat-miR-125b transfected with A20, T2 vector, T2-miR-125b, T2-miR-125b transfected with A20 cell lines were incubated with CD4 and CD8 antibodies for 45min, washed, and then the cells were analyzed by flow cytometry. bars represent SE. **, p<0.01.

Since A20 is a direct target of miR-125b and overexpression of miR-125b inhibits T cell differentiation, we next examined whether miR-125b regulates T cells differentiation through targeting A20. We first explored whether overexpression of A20 induces the differentiation of T cells. We forced overexpression of A20 into Jurkat cells. Compared to cells transfected with control vector, A20 overexpressing cells have an increased population of CD4+ cells (Figure 3A). To further confirm this result, we knocked down the expression of A20 in Jurkat cells by two different siRNAs. Depletion of A20 decreased the expression of CD4 in a dosage-dependent manner (Figure 3B). To test whether miR-125b regulates the expression CD4 through A20, we restored the expression of A20 in miR-125b overexpressing cells (Jurkat-miR-125b and T2-miR-125b) with an A20 expressing vector pMSCV-A20 (Figure 3C and 3D, Supplementary Figure 2). Restoration of A20 in miR-125b overexpressing cells dramatically decreased CD4- cells to the basal percentage of CD4- cell population in the control cells (Jurkat-vector and T2 vector cells). These results support that miR-125b regulates T cell differentiation through targeting A20.

### Inhibition of T cell differentiation by miR-125b does not depend on NF-kB

**Figure 4:**
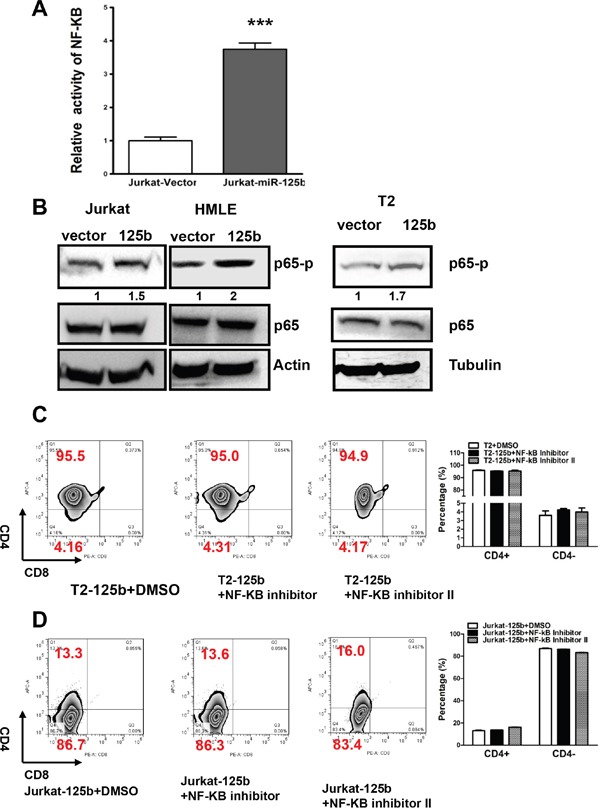
miR-125b inhibition of the differentiation of T cells is NF-kB independent **A.** Jurkat-vector and Jurkat-miR-125b cells were transfected with NF-κB reporter by using Lipofectamine 2000 reagent. 48 hours post-transfection, cells were harvested and lysed with passive lysis buffer. Luciferase activity was measured by using a dual luciferase reporter assay. The pRL-TK vector was used as an internal control. The results were expressed as relative luciferase activity (firefly LUC/Renilla LUC). **B.** Jurkat-vector, Jurkat-A20, T2-vector, T2 –miR-125b, HMLE-vector and HMLE-miR-125b stable cell lines were collected. Cell lysates were prepared for Western blotting with antibodies against p65 and p65-p. β-actin was used as a loading control. **C** and **D.** Jurkat-miR-125b and T2-miR-125b cells were treated with DMSO (control) and NF-κB inhibitors (Calbiochem) for 48 hrs. Cells were incubated with CD4 and CD8 antibodies for 45 min, washed, and then the cells were analyzed by flow cytometry.

It has been known that A20 negatively regulates inflammation by inhibiting NF-kB and NF-kB plays an important role in T cell differentiation [21]. We next examined whether miR-125b regulates T cell differentiation also through NF-kB signaling pathway. First we tested whether overexpression of miR-125b activates NF-kB. We transfected NF-kB reporter into Jurkat-vector and Jurkat-miR-125b cells (Figure 4A). Compared to cells transfected with control vector, miR-125b overexpressing cells have much higher activity of NF-kB. To further confirm this result, we detected the phosphorylation level of p65, an indicator of NF-kB activation, in cells with overexpressing miR-125b and control cells by immunoblotting. Compared to cells transfected with control vector, miR-125b overexpressing cells showed much higher phosphorylation level of p65 (Figure 4B). These results further confirmed that miR-125b increases the activity of NF-kB. To examine whether inhibition of NF-kB activity induces the differentiation of T-ALL cells, we treated T2-miR-125b and Jurkat-miR-125b cells with control (DMSO) or two different NF-kB inhibitors. We found that inhibition of NF-kB did not block miR-125b induced T-ALL cell dedifferentiation (Figure 4C and 4D, Supplementary Figure 3). These results support that miR-125b inhibits the differentiation of T cell and this process is independent of NF-kB activity.

### miR-125b regulates the glucose metabolism in T cells via A20

**Figure 5:**
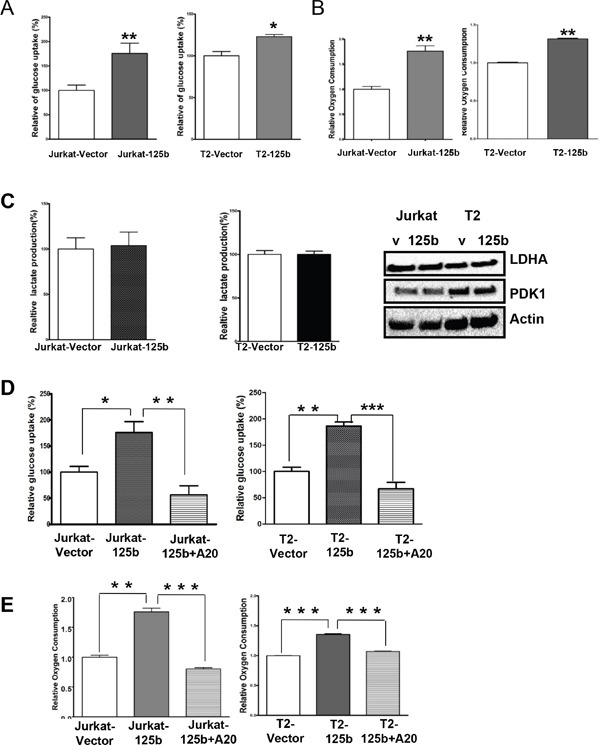
miR-125b regulates the glucose metabolism in T cells via A20 **A** and **D.** Cells were cultured in medium containing 10% fetal bovine serum (FBS) and glucose uptake was measured. Data are showing percentage relative to Jurkat –vector (left) or T2 vector (right). **B** and **E.** Oxygen consumption rates of Jurakt-vector, Jurkat-miR-125b, Jurkat-miR-125b transfected with A20 (left), T2-vector and T2-miR-125b Jurkat-miR-125b transfected with A20 cells (right) were measured. The oxygen consumption rate was calculated on the basis of the maximal rate of change in relative fluorescence units (DFU/second). Columns, mean of three independent experiments **C.** Cells were cultured in medium containing10%FBS and lactate production in the medium was measured as described in Materials and methods. Data are showing percentage. Jurkat-vector, Jurkat-A20, T2-vector and T2 –miR-125b stable cell lines were collected. Cell lysates were prepared for Western blotting with antibodies against PDK1 and LDHA. β-actin was used as a loading control.

It has been known that glucose metabolism regulates T cell differentiation [22]. Since T cell differentiation is regulated by miR-125b, we surmised that miR-125b may also regulate glucose metabolism in T cell leukemia. To examine whether the overexpression of miR-125b alters glucose metabolism in human T cells, glucose uptake, oxygen consumption and lactate production, which are hallmarks of glycolysis, were measured and compared in vector and miR-125b-overexpressing cells. Both Jurkat-miR-125b and T2-miR-125b cells showed a significantly higher glucose uptake (Figure 5A) and oxygen consumption (Figure 5B) compared to Jurkat-vector and T2-vector, respectively. However, there was no change of lactate production in these two pairs (Figure 5C). To further confirm the lactate production, we examined the LDHA protein expression level in miR-125b overexpressing and control cells by immunoblotting. The result showed there was also no change of LDHA expression level between vector cells and miR-125b overexpressing cells (Figure 5C). Since miR-125b regulates T cell differentiation through A20, we deduced that miR-125b may also regulate glucose metabolism through A20. To test the hypothesis, we first explored whether A20 alters glucose metabolism in human T cells, we knocked down A20 in Jurkat cells with siRNA, and measured glucose uptake. Knockdown of A20 in Jurkat cells increased glucose uptake in comparison with scrambled siRNA (Supplementary Figure 4). We restored the expression of A20 in miR-125b overexpressing cells with pMSCV-A20 and examined the glucose uptake and oxygen consumption. Restoration of A20 in miR-125b overexpressing cells dramatically decreased oxygen consumption and glucose uptakes to the level of cells transfected with empty vector (Figure 5D and 5E). These results indicate that A20 is a critical mediator of miR-125b function in enhancing glucose uptake and oxygen consumption.

## DISCUSSION

Many miRNAs have been identified as having a tumor suppressive or oncogenic function [12]. Several miRNAs have been reported to regulate T cell differentiation [23]. T cell development is a strictly regulated process from hematopoietic progenitor cells to early T cell progenitors, and then toward mature and functional T cells [3]. Chromosomal and gene aberrations drive the immature thymocytes into uncontrolled clonal expansion and cause T-cell acute lymphoblastic leukemia (T-ALL) [4, 24–27]. miR-125b is highly expressed in hematopoietic stem cells [19] and deregulation of miR-125b in hematopoietic cells results in leukemia [18, 20, 28–30]. A recent report shows that overexpression of miR-125b in early hematopoietic stem/progenitor cells leads to leukemia in mice, and about 50 percent of the mice developed T-cell acute lymphoblastic leukemia (T-ALL) [20]. However, how miR-125b causes T-ALL is still not clear.

In this study, we have showed that miR-125b regulates T cell differentiation into CD4+ cells and metabolic reprogramming via A20. We found that compared to CD4+ cells, miR-125b is up-regulated in CD4- Jurkat cells. Overexpression of miR-125b blocked CD4+ T cell differentiation. Mutation and deletion of A20 which inhibits NF-kB is found in leukemia [10, 31–33]. Interestingly, B cells lacking A20 also display impaired differentiation [11]. Here we demonstrate that A20 is a direct target of miR-125b in T cells. Moreover, overexpression of A20 induced Jurkat cell differentiation into CD4+ cells. However, we found the impact of miR-125b on T cell differentiation is NF-kB independent. It has been reported that A20 also regulates the pro-apoptotic mitogen-activated protein kinase c-Jun N-terminal kinase (JNK), and a v-akt murine thymoma viral oncogene homolog (Akt). Since both JNK and Akt play a role in T cell differentiation [34–36], A20 may regulate T cell via AKt or JNK in our cells.

It has been reported that metabolic reprogramming induces cell survival and drug resistance [37–39]. Since glucose metabolism regulates T cell differentiation, we speculated that miR-125b may regulate glucose metabolism of T cells. Overexpression of miR-125b increased both glucose uptake and oxygen consumption of T cells but did not increase lactate production. These results indicate that miR-125b-induced deregulation of glucose metabolism is not a typical Warburg effect. However, metabolic plasticity and metabolic heterogeneity have been found in some cancers with aggressive phenotype [40–46]. It also has been reported that aerobic glycolysis is less active in T-ALL cells than normal T cells. Oncogenic Notch promoted glycolysis but also induced metabolic stress that activated AMP-activated kinase (AMPK) to promote oxidative metabolism and mitochondrial Complex I activity [47]. The increased glucose uptake of T cell leukemia cells combines with increased mitochondrial function to produce more ATP through the TCA cycle rather than aerobic glycolysis. To our knowledge, this is the first report to demonstrate miR-125b induces glucose metabolic switch in T cell via targeting A20. We found overexpression of miR-125b increased the expression of glut1 in Jurkat and T2 cells while overexpression of A20 in Jurkat cells decreased the expression of Glut1 (Data not shown). The data suggest that miR-125b regulates glucose uptake via glut1 by reducing the expression of A20. But we found inhibition of glucose uptake by 2-DG or glucose transporter glut1 inhibitor (WZB117) did not induce CD4 T cell differentiation (data not shown). These results suggest glucose metabolism and T cell differentiation in leukemia do not necessarily depend on each other, which are different from normal T cell differentiation [20].

Significantly, our novel findings have important implications in understanding the mechanism of T-ALL development. Moreover, since both de-differentiation and dysregulated glucose metabolism contribute to the development of T-cell leukemia, simultaneously targeting de-differentiation process and inhibiting dysregulated glucose metabolism may serve as an effective therapeutic strategy for treating miR-125b expressing T cell leukemia.

## MATERIALS AND METHODS

### Cells and cell culture

Human T lymphocytic leukemia cell lines, Jurkat and T2 cell lines were purchased from American Type Culture Collection (ATCC). Cells were cultured in RPMI-1640 (Mediatech Inc.) supplemented with 10% FBS and Penicillin/Streptomycin. HMLE (immortalized mammary epithelial cells) cell line was cultured in 1:1 Dulbecco's Modified Eagle's Medium (DMEM)/Ham's F-12 medium (Mediatech Inc) supplemented with 5% FBS (Clontech), 100 units/ml penicillin-streptomycin (Invitrogen), 2 mm L-glutamine (Invitrogen), 10 ng/ml human epidermal growth factor (EGF) (Invitrogen), 0.5 μg/ml hydrocortisone (Sigma), and 10 μg/ml insulin (Sigma).

### Flow cytometry analysis

Cells (1×10^6^) were incubated with CD4-APC and CD8-PE (BD Biosciences) conjugated antibodies and placed on ice 45 minutes, then washed with blocking buffer (PBS with 0.1% Na_2_N_3_, 1%FBS). CD4/CD8 markers were analyzed using a FACSCanto Flow Cytometer (BD Biosciences).

### Inhibition of NF-κB

Jurkat-miR-125b or T2-miR-125b cells (1×10^6^) were treated with NK-kB inhibitior (Calbiochem, quinazoline) and NK-kB inhibitor II (Calbiochem, 4-Methyl-N-(3-phenylpropyl) benzene-1, 2-diamine) for 48 hrs. Then cells were incubated with CD4-APC and CD8-PE, and CD4/CD8 markers were analyzed using a FACSCanto Flow Cytometer.

### Vector construction and establisment of stable cell lines

Full length human precursor miR-125b together with 150 bp of flanking sequence was amplified from human genomic DNA and cloned into pMSCVpuro expression vector (Clontech). Sponge was cloned into pMSCV-GFP. miR-125b sponge has 8 miR-125b binding sites as we reported [14]. A20 was amplified from T2 cDNA with primers: forward 5′-TGACTC-GAGATGGCTGAACAAGTCCTTCCT-3′; reverse, 5′-CTAGAATTCTC-AGCGGGGACATCCTGAG-3′, and cloned into pMSCVpuro (or neo) vector with XhoI and EcoRI and named pMSCV-A20. All cloned fragments were verified by sequencing. Infectious and replication-incompetent retroviral particles were produced according to manufacturer's protocol. Jurkat, T2 and HMLE cells were infected with retroviral particles expressing miR-125b or the puromycin resistance gene only (vector control). Jurkat and T2 cells were infected with retroviral particles expressing miR-125b spong1 and scramble. After retroviral infection and primary puromycin selection at 1μg/ml, cell pools of Jurkat-125b, Jurkat-vector, T2-125b, T2-vector and Jurkat-mir-125b-spong were maintained for multiple culture passages under puromycin concentration (1μg/ml). Plasmids were transfected with Lipofactamine 2000 (Invitrogen) according to manufacturer's protocol.

### Quantitative reverse transcription PCR (qRT-PCR)

Total RNA was isolated from cultured cells using TRIzol reagent (Invitrogen). For miRNA expression analysis, qRT-PCR was performed using qRT-PCR miRNA Detection Kit and mirVana qRT-PCR Primer Sets (Applied Biosystems) according to the manufacturer's protocols. Human U6 served as an internal control. For quantitative PCR, cDNA was mixed with 2 × SYBR Green PCR Master Mix (Applied Biosystems) and various sets of gene-specific primers and then subjected to RT-PCR quantification using the iQ5 Real-Time PCR system (Bio-Rad). All reactions were performed in triplicate. The relative amounts of mRNA were calculated by using the comparative Ct method. The results are presented as fold-change of each miRNA.

### A20 siRNA transfection

siRNA oligonucleotides for A20 were purchased from Sigma, with a scrambled siRNA (Sigma) serving as a control. Transfection was performed using Lipofectamine 2000 Transfection Reagent (Invitrogen) according to the manufacturer's protocol. Forty-eight hrs after transfection, the cell lysates were prepared for further analysis by Western blotting.

### Luciferase assay

To construct a reporter plasmid containing the 3′ UTR of A20, a 1.567 kb DNA fragment of the 3′ UTR of A20 was amplified from 293T genomic DNA with primers: forward 5′-CATACTAG-TCCGGAAACAGGTGGGTCACCT, reverse 5′-CTCGTTTAAACCTTCTAG-AGCAAATATGACAG, and cloned into the pMIR-REPORT Luciferase vector (named as A20Wt 3UTR) downstream of the Luciferase gene in a sense or antisense direction. A20Wt 3UTR with deletion of miR-125b binding site (named as A20 Del 3UTR) was amplified from A20Wt 3UTR with primers: GCAAGAAGCTCAAGGAA-GAAATGGACGTATTCAGAG; CTCT-GAATACGTCCA TTTCTTCCTTGAGCTTCTTGC. All PCR products cloned into the plasmid and the cloning directions were verified by DNA sequencing. For the luciferase assay, Jurkat cells were co-transfected with pMIR-REPORT luciferase reporters, miR-125b or miR-control. Two days later, cells were harvested and lysed with passive lysis buffer (Promega). Luciferase activity was measured by using a dual luciferase reporter assay (Promega). The pRL-tk Renilla luciferase plasmid (Promega) was used as an internal control. The results were expressed as relative luciferase activity (LUC/Renilla LUC).

### Glucose uptake assay

Cells were seeded in 24-well plates at 5~10 × 105 cells/well. Culture media were collected at 4 and 8h and stored at −20°C until they were assayed. Glucose uptake was measured using the Amplex Red Glucose/Glucose Oxidase Assay Kit (Molecular Probes, Carlsbad, CA, USA) or Glucose colorimetric/Fluorometric Assay Kit (BioVision, Mountain View, CA, USA). Absorbance was measured at 563 nm using a SpectraMax M5 plate reader (Molecular Devices, Sunnyvate, CA, USA) and results were normalized on the basis of the total protein amounts of the cells.

### Lactate production assay

Lactate production in the medium was detected by using the Lactate Assay Kit (BioVision, Mountain View, CA, USA). Results were normalized on the basis of the total protein amounts of the cells.

### O_2_ consumption assay

Cells were transferred to 96-well O_2_ biosensor plates (BD Biosciences, San Jose, CA, USA) with cyclodextran beads, at a density of 2 × 10^6^ cells/well. Fluorescence was measured at an excitation of 485nm and an emission of 630nm. Oxygen consumption rate was expressed as ΔFU per second.

### Western blotting

Cells were harvested and lysed in NETN (20 mM Tris-HCl, pH 8.0, 100 mM NaCl, 1 mM EDTA, 0.5% NP40) for 10 min on ice. Lysates were cleared by centrifugation at 13,200 rpm at 4°C for 10 min. Supernatants were collected and protein concentrations were determined by the Bradford assay (Bio-rad). The proteins were then separated with a SDS/polyacrylamide gel and transferred to a Nitrocellulose membrane (Bio-rad). After blocking in TBS with 5% BSA (Sigma) for 1 hr, the membranes were incubated overnight at 4-8°C with the primary antibodies in TBST containing 1% BSA. The following antibodies were utilized: the β-actin antibody was purchased from Sigma. The tubulin and A20 antibodies were purchased from Santa Cruz. LDHA, PDK1, P65 and p65-p antibodies were purchased from Cell Signaling. Membranes were extensively washed with TBST buffer and incubated with horseradish peroxidase conjugated secondary anti-mouse antibody or anti-rabbit antibody (dilution 1:2,500, BioRad). After additional washes with TBST buffer, antigen-antibody complexes were visualized with the enhanced chemiluminescence kit (Pierce).

### Cell viability assay

A total of 5 × 10^3^~1 × 10^4^ cells/well were seeded in 96-well plates. Twenty-four hrs later, the medium was replaced with fresh medium and then incubated for 48 hrs. Cell viability was determined using CellTiter 96 AQueous One Solution Cell Proliferation Assay kit (Promega).

### Statistical analysis

Statistical evaluation for data analysis was determined by Unpaired Student's t-test. All data is shown as the mean ± standard error (SE). *P* < 0.05 was considered statistically significant.

## SUPPLEMENTARY FIGURES



## References

[1] Pui CH, Evans WE (2006). Treatment of acute lymphoblastic leukemia. N Engl J Med.

[2] Asnafi V, Buzyn A, Le Noir S, Baleydier F, Simon A, Beldjord K, Reman O, Witz F, Fagot T, Tavernier E, Turlure P, Leguay T, Huguet F (2009). NOTCH1/FBXW7 mutation identifies a large subgroup with favorable outcome in adult T-cell acute lymphoblastic leukemia (T-ALL): a Group for Research on Adult Acute Lymphoblastic Leukemia (GRAALL) study. Blood.

[3] Peirs S, Van der Meulen J, Van de Walle I, Taghon T, Speleman F, Poppe B, Van Vlierberghe P (2015). Epigenetics in T-cell acute lymphoblastic leukemia. Immunol Rev.

[4] Liu H, Chiang MY, Pear WS (2011). Critical roles of NOTCH1 in acute T-cell lymphoblastic leukemia. Int J Hematol.

[5] Mets E, Van der Meulen J, Van Peer G, Boice M, Mestdagh P, Van de Walle I, Lammens T, Goossens S, De Moerloose B, Benoit Y, Van Roy N, Clappier E, Poppe B (2015). MicroRNA-193b-3p acts as a tumor suppressor by targeting the MYB oncogene in T-cell acute lymphoblastic leukemia. Leukemia.

[6] Wertz IE, O'Rourke KM, Zhou H, Eby M, Aravind L, Seshagiri S, Wu P, Wiesmann C, Baker R, Boone DL, Ma A, Koonin EV, Dixit VM (2004). De-ubiquitination and ubiquitin ligase domains of A20 downregulate NF-kappaB signalling. Nature.

[7] Shembade N, Harhaj EW (2012). Regulation of NF-kappaB signaling by the A20 deubiquitinase. Cell Mol Immunol.

[8] Catrysse L, Vereecke L, Beyaert R, van Loo G (2014). A20 in inflammation and autoimmunity. Trends Immunol.

[9] Kato M, Sanada M, Kato I, Sato Y, Takita J, Takeuchi K, Niwa A, Chen Y, Nakazaki K, Nomoto J, Asakura Y, Muto S, Tamura A (2009). Frequent inactivation of A20 in B-cell lymphomas. Nature.

[10] Johansson P, Bergmann A, Rahmann S, Wohlers I, Scholtysik R, Przekopowitz M, Seifert M, Tschurtschenthaler G, Webersinke G, Jager U, Siebert R, Klein-Hitpass L, Duhrsen U (2016). Recurrent alterations of TNFAIP3 (A20) in T-cell large granular lymphocytic leukemia. Int J Cancer.

[11] Chu Y, Vahl JC, Kumar D, Heger K, Bertossi A, Wojtowicz E, Soberon V, Schenten D, Mack B, Reutelshofer M, Beyaert R, Amann K, van Loo G (2011). B cells lacking the tumor suppressor TNFAIP3/A20 display impaired differentiation and hyperactivation and cause inflammation and autoimmunity in aged mice. Blood.

[12] Lin S, Gregory RI (2015). MicroRNA biogenesis pathways in cancer. Nat Rev Cancer.

[13] Zhou M, Liu Z, Zhao Y, Ding Y, Liu H, Xi Y, Xiong W, Li G, Lu J, Fodstad O, Riker AI, Tan M (2010). MicroRNA-125b confers the resistance of breast cancer cells to paclitaxel through suppression of pro-apoptotic Bcl-2 antagonist killer 1 (Bak1) expression. J Biol Chem.

[14] Liu Z, Liu H, Desai S, Schmitt DC, Zhou M, Khong HT, Klos KS, McClellan S, Fodstad O, Tan M (2013). miR-125b functions as a key mediator for snail-induced stem cell propagation and chemoresistance. J Biol Chem.

[15] Zhang L, Ge Y, Fuchs E (2014). miR-125b can enhance skin tumor initiation and promote malignant progression by repressing differentiation and prolonging cell survival. Genes Dev.

[16] Liu LH, Li H, Li JP, Zhong H, Zhang HC, Chen J, Xiao T (2011). miR-125b suppresses the proliferation and migration of osteosarcoma cells through down-regulation of STAT3. Biochem Biophys Res Commun.

[17] Gururajan M, Haga CL, Das S, Leu CM, Hodson D, Josson S, Turner M, Cooper MD (2010). MicroRNA 125b inhibition of B cell differentiation in germinal centers. Int Immunol.

[18] Bousquet M, Quelen C, Rosati R, Mansat-De Mas V, La Starza R, Bastard C, Lippert E, Talmant P, Lafage-Pochitaloff M, Leroux D, Gervais C, Viguie F, Lai JL (2008). Myeloid cell differentiation arrest by miR-125b-1 in myelodysplastic syndrome and acute myeloid leukemia with the t(2;11)(p21;q23) translocation. J Exp Med.

[19] Ooi AG, Sahoo D, Adorno M, Wang Y, Weissman IL, Park CY (2010). MicroRNA-125b expands hematopoietic stem cells and enriches for the lymphoid-balanced and lymphoid-biased subsets. Proc Natl Acad Sci U S A.

[20] Bousquet M, Harris MH, Zhou B, Lodish HF (2010). MicroRNA miR-125b causes leukemia. Proc Natl Acad Sci U S A.

[21] Gerondakis S, Fulford TS, Messina NL, Grumont RJ (2014). NF-kappaB control of T cell development. Nat Immunol.

[22] Palmer CS, Ostrowski M, Balderson B, Christian N, Crowe SM (2015). Glucose metabolism regulates T cell activation, differentiation, and functions. Front Immunol.

[23] Jeker LT, Bluestone JA (2013). MicroRNA regulation of T-cell differentiation and function. Immunol Rev.

[24] Evangelisti C, Chiarini F, Lonetti A, Buontempo F, Bressanin D, Cappellini A, Orsini E, McCubrey JA, Martelli AM (2014). Therapeutic potential of targeting mTOR in T-cell acute lymphoblastic leukemia (review). Int J Oncol.

[25] Van Vlierberghe P, Ferrando A (2012). The molecular basis of T cell acute lymphoblastic leukemia. J Clin Invest.

[26] Hagemeijer A, Graux C (2010). ABL1 rearrangements in T-cell acute lymphoblastic leukemia. Genes Chromosomes Cancer.

[27] De Keersmaecker K (2008). ABL1 fusions in T-cell acute lymphoblastic leukemia. Verh K Acad Geneeskd Belg.

[28] Chaudhuri AA, So AY, Mehta A, Minisandram A, Sinha N, Jonsson VD, Rao DS, O'Connell RM, Baltimore D (2012). Oncomir miR-125b regulates hematopoiesis by targeting the gene Lin28A. Proc Natl Acad Sci U S A.

[29] Surdziel E, Cabanski M, Dallmann I, Lyszkiewicz M, Krueger A, Ganser A, Scherr M, Eder M (2011). Enforced expression of miR-125b affects myelopoiesis by targeting multiple signaling pathways. Blood.

[30] Gefen N, Binder V, Zaliova M, Linka Y, Morrow M, Novosel A, Edry L, Hertzberg L, Shomron N, Williams O, Trka J, Borkhardt A, Izraeli S (2010). Hsa-mir-125b-2 is highly expressed in childhood ETV6/RUNX1 (TEL/AML1) leukemias and confers survival advantage to growth inhibitory signals independent of p53. Leukemia.

[31] Paik JH, Go H, Nam SJ, Kim TM, Heo DS, Kim CW, Jeon YK (2013). Clinicopathologic implication of A20/TNFAIP3 deletion in diffuse large B-cell lymphoma: an analysis according to immunohistochemical subgroups and rituximab treatment. Leuk Lymphoma.

[32] Schmitz R, Hansmann ML, Bohle V, Martin-Subero JI, Hartmann S, Mechtersheimer G, Klapper W, Vater I, Giefing M, Gesk S, Stanelle J, Siebert R, Kuppers R (2009). TNFAIP3 (A20) is a tumor suppressor gene in Hodgkin lymphoma and primary mediastinal B cell lymphoma. J Exp Med.

[33] Honma K, Tsuzuki S, Nakagawa M, Tagawa H, Nakamura S, Morishima Y, Seto M (2009). TNFAIP3/A20 functions as a novel tumor suppressor gene in several subtypes of non-Hodgkin lymphomas. Blood.

[34] Fukaya M, Brorsson CA, Meyerovich K, Catrysse L, Delaroche D, Vanzela EC, Ortis F, Beyaert R, Nielsen LB, Andersen ML, Mortensen HB, Pociot F, van Loo G (2016). A20 Inhibits beta-Cell Apoptosis by Multiple Mechanisms and Predicts Residual beta-Cell Function in Type 1 Diabetes. Mol Endocrinol.

[35] Kim EH, Suresh M (2013). Role of PI3K/Akt signaling in memory CD8 T cell differentiation. Front Immunol.

[36] Dong C, Yang DD, Tournier C, Whitmarsh AJ, Xu J, Davis RJ, Flavell RA (2000). JNK is required for effector T-cell function but not for T-cell activation. Nature.

[37] Staubert C, Bhuiyan H, Lindahl A, Broom OJ, Zhu Y, Islam S, Linnarsson S, Lehtio J, Nordstrom A (2015). Rewired metabolism in drug-resistant leukemia cells: a metabolic switch hallmarked by reduced dependence on exogenous glutamine. J Biol Chem.

[38] Herranz D, Ambesi-Impiombato A, Sudderth J, Sanchez-Martin M, Belver L, Tosello V, Xu L, Wendorff AA, Castillo M, Haydu JE, Marquez J, Mates JM, Kung AL (2015). Metabolic reprogramming induces resistance to anti-NOTCH1 therapies in T cell acute lymphoblastic leukemia. Nat Med.

[39] Coloff JL, Mason EF, Altman BJ, Gerriets VA, Liu T, Nichols AN, Zhao Y, Wofford JA, Jacobs SR, Ilkayeva O, Garrison SP, Zambetti GP, Rathmell JC (2011). Akt requires glucose metabolism to suppress puma expression and prevent apoptosis of leukemic T cells. J Biol Chem.

[40] Xu XD, Shao SX, Jiang HP, Cao YW, Wang YH, Yang XC, Wang YL, Wang XS, Niu HT (2015). Warburg effect or reverse Warburg effect? A review of cancer metabolism. Oncol Res Treat.

[41] Zhang G, Li J, Wang X, Ma Y, Yin X, Wang F, Zheng H, Duan X, Postel GC, Li XF (2015). The reverse Warburg effect and 18F-FDG uptake in non-small cell lung cancer A549 in mice: a pilot study. J Nucl Med.

[42] Sotgia F, Martinez-Outschoorn UE, Lisanti MP (2014). The reverse Warburg effect in osteosarcoma. Oncotarget.

[43] Liu W, Beck BH, Vaidya KS, Nash KT, Feeley KP, Ballinger SW, Pounds KM, Denning WL, Diers AR, Landar A, Dhar A, Iwakuma T, Welch DR (2014). Metastasis suppressor KISS1 seems to reverse the Warburg effect by enhancing mitochondrial biogenesis. Cancer Res.

[44] Martinez-Outschoorn UE, Whitaker-Menezes D, Valsecchi M, Martinez-Cantarin MP, Dulau-Florea A, Gong J, Howell A, Flomenberg N, Pestell RG, Wagner J, Arana-Yi C, Sharma M, Sotgia F (2013). Reverse Warburg effect in a patient with aggressive B-cell lymphoma: is lactic acidosis a paraneoplastic syndrome?. Semin Oncol.

[45] Witkiewicz AK, Whitaker-Menezes D, Dasgupta A, Philp NJ, Lin Z, Gandara R, Sneddon S, Martinez-Outschoorn UE, Sotgia F, Lisanti MP (2012). Using the “reverse Warburg effect” to identify high-risk breast cancer patients: stromal MCT4 predicts poor clinical outcome in triple-negative breast cancers. Cell Cycle.

[46] Pavlides S, Whitaker-Menezes D, Castello-Cros R, Flomenberg N, Witkiewicz AK, Frank PG, Casimiro MC, Wang C, Fortina P, Addya S, Pestell RG, Martinez-Outschoorn UE, Sotgia F (2009). The reverse Warburg effect: aerobic glycolysis in cancer associated fibroblasts and the tumor stroma. Cell Cycle.

[47] Kishton RJ, Barnes CE, Nichols AG, Cohen S, Gerriets VA, Siska PJ, Macintyre AN, Goraksha-Hicks P, de Cubas AA, Liu T, Warmoes MO, Abel ED, Yeoh AE (2016). AMPK Is Essential to Balance Glycolysis and Mitochondrial Metabolism to Control T-ALL Cell Stress and Survival. Cell Metab.

