# Supplementary material for: miR-125b regulates differentiation and metabolic reprogramming of T cell acute lymphoblastic leukemia by directly targeting A20
Source: Oncotarget. 2016 Sep 14;7(48):78667–79. doi: 10.18632/oncotarget.12018 (PMC5346668; doi:10.18632/oncotarget.12018)
Supplement: Supplementary file 1 [file oncotarget-07-78667-s001.pdf]

miR-125b regulates differentiation and metabolic reprogramming of T cell acute lymphoblastic leukemia by directly targeting A20

SUPPLEMENTARY FIGURES

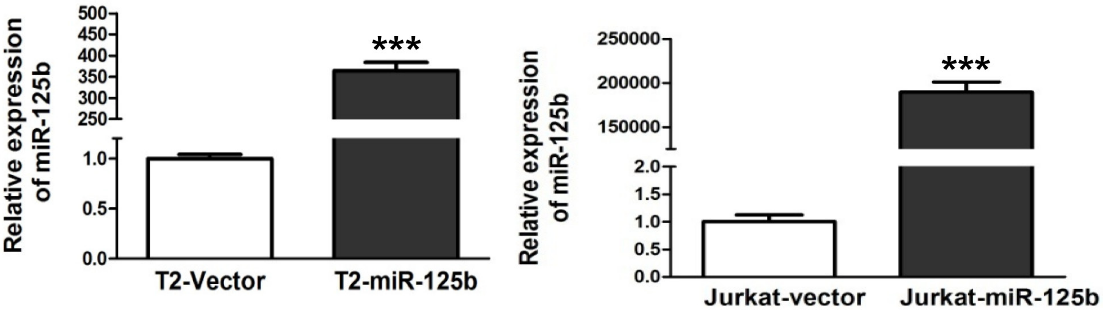

Supplementary Figure S1: Quantitative Real-time PCR (qRT-PCR)—Total RNA was isolated from cultured cells using TRIzol reagent (Invitrogen). For miRNA expression analysis, qRT-PCR was performed using qRT-PCR miRNA Detection Kit and mirVana qRT-PCR Primer Sets (Applied Biosystems) according to the manufacturer's protocols. Human U6 served as an internal control. For quantitative PCR, cDNA was mixed with 2 SYBR Green PCR Master Mix (Applied Biosystems) and various sets of gene-specific primers and then subjected to RT-PCR quantification using the iQ5 Real-Time PCR system (Bio-Rad). All reactions were performed in triplicate. The relative amounts of mRNA were calculated by using the comparative CT method. The results are presented as fold change of each miRNA.

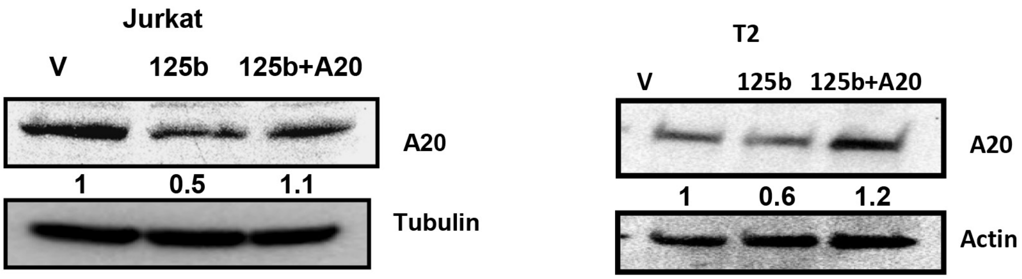

Supplementary Figure S2: Jurkat-vector, Jurkat-miR-125b, Jurkat-miR-125b transfected with A20, T2 vector, T2-miR-125b and T2-miR-125b transfected with A20 cells were collected. Cell lysates were prepared for Western blotting with an antibody against A20, and β-actin was used as a loading control.

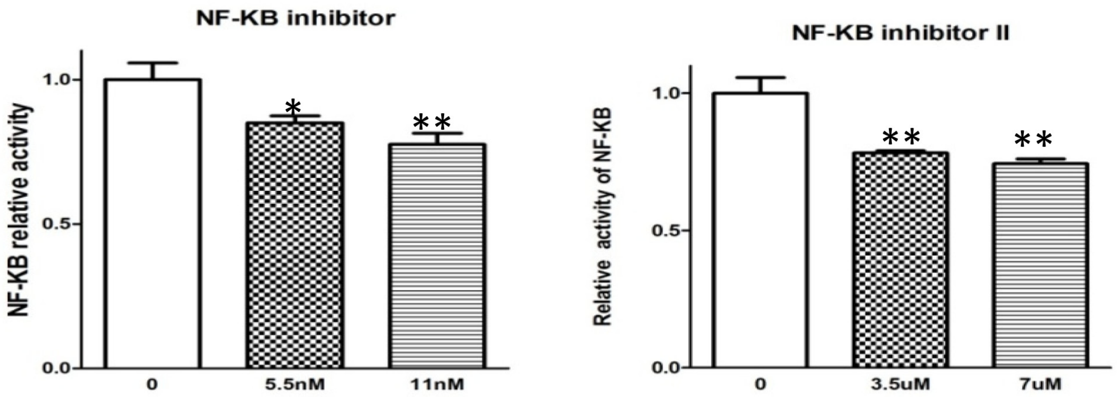

Supplementary Figure S3: Jurkat-miR-125b cells were cotransfected with NF-kB-luciferase reporter and pRL-Tk by Lonza Nucleofector. Cells were treated with NF-kB inhibitors for 48 hrs pRL-TK was used as an internal control. Each transfection was performed in triplicates. Luciferase activity was measured by using a dual luciferase reporter assay (Promega) as manufacturer's protocol. Columns, mean of three independent experiments; bars, SE. *, P<0.05. bars, SE. **, P<0.01.

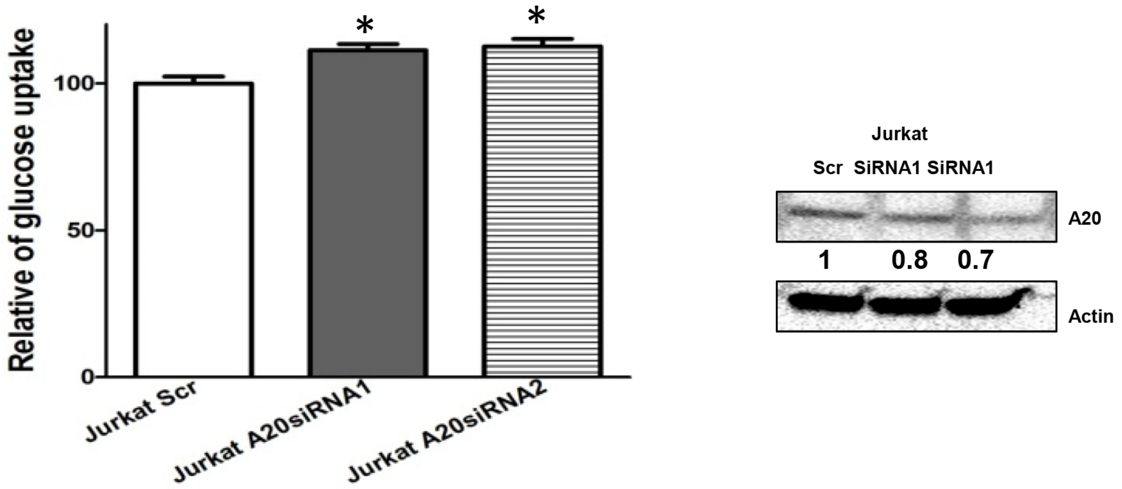

Supplementary Figure S4: Jurkat cells were transfected with A20 siRNAs and scrambled siRNA according to the manufacturer's protocol. Forty-eight hrs after transfection, Cells were seeded in 24-well plates at 5~10 × 105 cells/well. Culture media were collected at 4 and 8h and stored at −20°C until they were assayed. Glucose uptake was measured using Glucose colorimetric/Fluorometric Assay Kit (BioVision, Mountain View, CA, USA). Absorbance was measured at 563 nm using a SpectraMax M5 plate reader (Molecular Devices, Sunnyvate, CA, USA) and results were normalized on the basis of the total protein amounts of the cells. Cell lysates were prepared for Western blotting with an antibody against A20, and β-actin was used as a loading control.
